# Supplementary material for: OpenMM 7: Rapid development of high performance algorithms for molecular dynamics
Source: PLoS Comput Biol. 2017 Jul 26;13(7):e1005659. doi: 10.1371/journal.pcbi.1005659 (PMC5549999; doi:10.1371/journal.pcbi.1005659)
Supplement: S1 Source Code — (ZIP) [file pcbi.1005659.s002.zip › OpenMM-7.0.1-Source/docs/api-c++/generated/OpenMM.OpenMMException.html]

OpenMMException — OpenMM @OPENMM\_MAJOR\_VERSION@.@OPENMM\_MINOR\_VERSION@ documentation


# OpenMMException¶

*class* `OpenMM::``OpenMMException`¶
:   This class is used for all exceptions thrown by OpenMM.

    Methods

    |  |  |
    | --- | --- |
    | `OpenMMException` |  |
    | `~OpenMMException` |  |
    | `what` |  |

    `OpenMMException`(*const* std::string &*message*)¶

    `~OpenMMException`()¶

    *const* char \*`what`() *const*¶

High performance molecular simulation on GPUs


### Navigation

- Core Objects
- Forces
- Integrators
- Extras

©2015, Stanford University and the Authors.
|
Powered by Sphinx 1.3.1
& Alabaster 0.7.7
|
Page source
